# Glucose Content and In Vitro Bioaccessibility in Sweet Potato and Winter Squash Varieties during Storage

**DOI:** 10.3390/foods6070048

**Published:** 2017-06-30

**Authors:** Fernanda Zaccari, María Cristina Cabrera, Ali Saadoun

**Affiliations:** 1 Poscosecha de Frutas y Hortalizas, Facultad de Agronomía, Universidad de la República, Av. Eugenio Garzón 780, CP12900 Montevideo, Uruguay; 2 Nutrición y Calidad de Alimentos, Facultad de Agronomía, Universidad de la República, Av. Eugenio Garzón 780, CP12900 Montevideo, Uruguay; mcab@fagro.edu.uy; 3 Fisiología y Nutrición, Facultad de Ciencias, Universidad de la República, Iguá 2245, CP11400 Montevideo, Uruguay; asaadoun@fcien.edu.uy

**Keywords:** glucose, bioaccessibility, *Ipomoea batatas*, *Cucurbita* sp., postharvest

## Abstract

Glucose content and in vitro bioaccessibility were determined in raw and cooked pulp of Arapey, Cuabé, and Beauregard sweet potato varieties, as well as Maravilla del Mercado and Atlas winter squash, after zero, two, four, and six months of storage (14 °C, 80% relative humidity (RH)). The total glucose content in 100 g of raw pulp was, for Arapey, 17.7 g; Beauregard, 13.2 g; Cuabé, 12.6 g; Atlas, 4.0 g; and in Maravilla del Mercado, 4.1 g. These contents were reduced by cooking process and storage time, 1.1 to 1.5 times, respectively, depending on the sweet potato variety. In winter squash varieties, the total glucose content was not modified by cooking, while the storage increased glucose content 2.8 times in the second month. After in vitro digestion, the glucose content released was 7.0 times higher in sweet potato (6.4 g) than in winter squash (0.91 g) varieties. Glucose released by in vitro digestion for sweet potato stored for six months did not change, but in winter squashes, stored Atlas released glucose content increased 1.6 times. In conclusion, in sweet potato and winter squash, the glucose content and the released glucose during digestive simulation depends on the variety and the storage time. These factors strongly affect the supply of glucose for human nutrition and should be taken into account for adjusting a diet according to consumer needs.

## 1. Introduction

Sweet potatoes (*Ipomoea batatas*, L.) and winter squashes (*Cucurbita* sp.) are two of the most important starch vegetable crops in the world [1,2,3,4]. Both vegetables—originating in the regions of South and Central America and produced and widely consumed in others countries, particularly in parts of Asia, Sub-Saharan Africa, and the Pacific Islands—are necessary for human nutrition [2,3,4]. The principal components in the fresh roots of sweet potatoes and winter squash fruits are carbohydrates; these make up around 25–28% fresh basis weight for sweet potato pulp, and 5–7% in winter squash [3,4,5,6]. Starch is the predominant carbohydrate, making up 85–95% and 11–62 % of total dry matter for sweet potato and winter squash, respectively [3,4,5]. Both vegetables can be harvested during summer and autumn seasons. The type of starch reserves in these vegetables influence the time of the postharvest conservation, i.e., three months for a medium length of time, or eight months for a long time, depending on the variety and storage conditions [3,4,5]. The starch, in roots and fruit, is metabolized to simple sugars, such as glucose, and it is used to maintain their viability during the postharvest life [3,4,5,6,7]. The interaction of the varieties, handling during the growing crop, and harvest and storage conditions could change the physiological process in vegetables and, in addition, the process of cooking could directly impact the amount and bioaccessibility of glucose content and of other compositional components of the pulp [7,8,9,10,11]. In spite of the fact that glucose is the key energy for life, particularly for the brain [12,13,14], a strong relationship between glucose the development of some diseases, such as diabetes, hypertension, and cardiovascular diseases, has been reported [15]. Consequently, sweet potato roots and winter squash fruits largely used in South American crops are interesting as energy sources for children and the elderly, and for persons with chronic diseases such as diabetes. In this last case, an accurate knowledge about glucose content and how much is released during storage or cooking is necessary. For this reason, the aim of this work was to determine the total glucose content and the effect of the cooking process on sweet potato and winter squash varieties during the storage period. Additionally, the glucose release during in vitro digestion was determined in cooked pulp from all varieties and storage times.

## 2. Experimental Section

### 2.1. Plant Materials and Sample Preparation

Three sweet potato varieties (Arapey, Cuabé, and Beauregard) are local varieties obtained in a breeding program [16], and two varieties of winter squash, one, a hybrid between *Cucurbita maxima* and *Cucurbita moschata* (named Maravilla del Mercado, Sakata) and the other type, “butternut”, *Cucurbita moschata* (Atlas, Sakata), were used. The roots of sweet potatoes and fruits of winter squashes were harvested at the mature stage at the end of summer (April). All of them were maintained for two weeks in an open room under initial handling wound healing conditions. After this time, the roots and fruits were carried to the Postharvest Fruits and Vegetables Laboratory of the Faculty of Agronomy. Selected roots and fruits were stored in a cold room at 14 °C and 80% relative humidity (RH) for zero (harvest), two, four, and six months. They were kept in three randomized plots per variety and storage period with 5–8 roots of sweet potato (≈2 kg per plots) and 12–15 fruits of winter squash per plot (≈25 kg per plots). After each storage time, the fruits were washed with tap water and soft brushing, rinsed with distilled water, drained and dried with blotting paper. Five roots and fruits per plot with no visible defects were used. The equatorial central part of the roots and fruits were used. Butternut squash fruits are pear-shaped. Therefore, in this case, the slices were obtained within the equatorial zone between the stem and the start of the seed cavity of the fruit. The pieces were peeled and cut with a stainless steel knife in cubes with 5 cm and 3 cm side lengths for winter squash pulp or sweet potato, respectively. The cubes were kept in sealed bags in a freezer (−20 °C) until analysis; previously, half of them were cooked in an oven microwave (Kassel^®^, KS-MM20, Hamburg, Germany) at 800 W, with hot water (≈55 °C) at a ratio of 1:2 (weight pulp: volume water) for 6 min. The final cooked temperature in the pulp was measured (60–65 °C) and the cube had an edible, firm texture and flavor. This cooking process was tested in previous trials. The variables studied were determined in duplicate for each treatment plot following analysis methodologies described below.

### 2.2. Total Glucose Content in Raw and Cooked Pulp

Extraction of glucose, in raw and cooked pulp, was performed with 0.5 g of pulp in 8 mL of HCl (4 N) boiling for 2 h. The extraction was filtered and NaOH (2 N) was added to neutralize the filtered solution. Total glucose content was measured by colorimetric methods using a commercial enzymatic procedure from Spinreacts kits (Glucose–TR, GOD-PROD, Sant Esteves de Bas, Girona, Spain). Determinations were obtained on a visible spectrophotometer (Genesys 10 VIS; Thermo Electro Corporation, Berlin, Germany) at *λ* = 505 nm. Data were expressed in grams of glucose per 100 g fresh weight (g 100 g^−1^ fw).

### 2.3. In Vitro Digestion of Cooked Pulp

An in vitro model based on a simulation digestion was performed. Samples of cooked pulp from sweet potato and winter squash varieties from every storage time were digested as described by Zaccari et al. [17]. Minor modifications was performed for the duodenal phase, including the addition of 0.1 mL α-amylase (A3306 Sigma-Aldrich, Saint Louis, MO, USA) and 30 μL α-amyloglucosidase (AT7095 Sigma-Aldrich, Saint Louise, MO, USA) at pH 6, and previously adjusting the digests with citrate buffer solution (pH 5.5) and NaHCO_3_ (0.8 M). After in vitro digestion, the digests were filtered and the glucose content in the extraction was measured.

### 2.4. In Vitro Bioaccessible Glucose Content in Cooked Pulp

The glucose content in the extracted digests was measured with similar procedures as those described for the total glucose measurement in pulp. Data were expressed in grams of total glucose released by in vitro digestion per 100 g cooked pulp weight (g 100 g^−1^ fw), and the percentage of bioaccessible glucose was calculated as:

(1) $$\%\;\mathrm{glucose}\;\mathrm{bioaccessible}=\frac{\mathrm{glucose}\;\mathrm{released}\;\mathrm{in}\;\mathrm{vitro}\;\mathrm{digestion}}{\mathrm{total}\;\mathrm{glucose}\;\mathrm{in}\;\mathrm{cooked}\;\mathrm{pulp}}\times 100$$

### 2.5. Experimental Design and Statistical Analysis

The experimental design had completely randomized plots (*n* = 3), in a 4 × 2 × 3 or 2 with factorial structure, storage factor with four storage times (zero, two, four, and six months), two preparation processes (raw or cooked), with three or two varieties of sweet potato or winter squash, respectively. For sweet potatoes and winter squash, data were analyzed by a three way-ANOVA (*p* ≤ 0.05) with variables including varieties, preparation, and storage times. Each preparation process was analyzed by one way-ANOVA (*p* ≤ 0.05) for sweet potato or winter squash, for varieties and storage time, followed by a Tukey post-hoc test (*p* ≤ 0.05). The effects of the preparation (raw and cooked) on each storage time and variety were analyzed by Student’s *t* test (*p* ≤ 0.05). The data was processed with the InfoStat (Version 2015; FCA, Córdoba, Argentine) statistical program. All values were presented as means ± SEM and expressed per 100 g of fresh pulp weight (100 g^−1^ fw).

## 3. Results and Discussion

### 3.1. Total Glucose Content in Raw and Cooked Pulp

**Figure 1:**
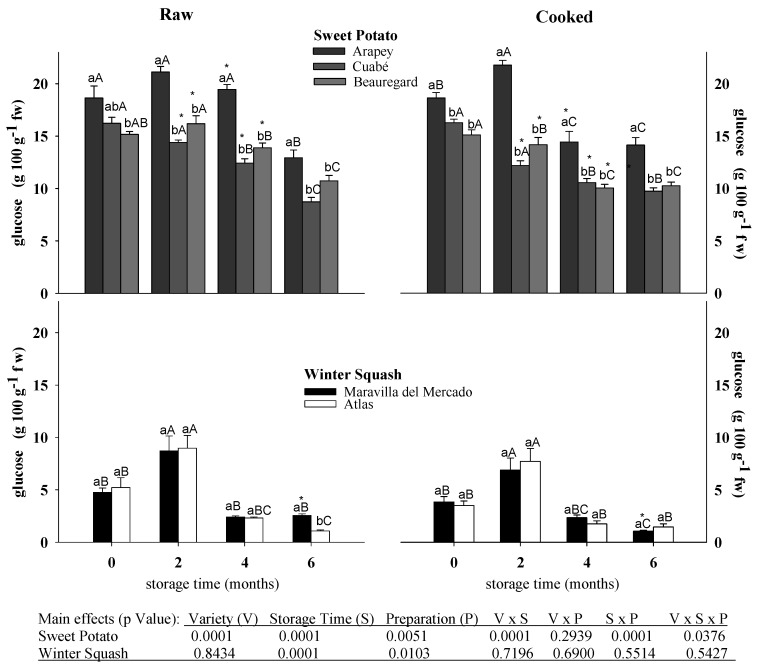
Total glucose content (g 100 g^−1^ fw) in raw and cooked pulp from sweet potato and winter squash varieties stored for different times. Means ± SEM (*n* = 6). Different lowercase letters on each column, for sweet potatoes or winter squash, indicate statistical differences (Tukey, *p* ≤ 0.05) between varieties for each process (raw or cooked) and storage time. Uppercase letters indicate statistical differences between storage times for each variety and process (raw or cooked). * denotes statistical differences by Student’s *t* test (*p* ≤ 0.05) between process (raw and cooked) in each variety and storage time.

The total glucose content in raw and cooked pulp was 3–4-fold higher in sweet potato (Arapey, 17.7 g; Beauregard, 13.2 g; Cuabé, 12.6 g) than in winter squash varieties (Atlas, 4.0 g; Maravilla del Mercado, 4.1 g) (Figure 1). In sweet potato, the total content of glucose depended on the interaction of the variety, method of preparation, and storage time; it decreased with the cooking process and times of storage (Figure 1). In sweet potato, raw and cooked pulp, Arapey was the variety that had the greatest total glucose content during four months of storage, and it decreased around 26% at six months. Furthermore, Beauregard and Cuabé varieties had similar glucose content (14.1 g) until the second month of storage, after which it decreased (10.3 g). The cooking process determined the reduction of the total glucose in the three varieties of sweet potatoes, especially at the second and fourth months of storage in Cuabé and Beauregard varieties (Figure 1).

However, in winter squash pulp, the total glucose content was similar in both varieties (4.0 g) and depended on the preparation and storage time (Figure 1). In raw and cooked pulp, it was observed that the total glucose content increased around 50–55% in the second month of storage. The raw pulp of winter squash had more (4.5 g) glucose content than cooked pulp (3.6 g). The effects of the cooking process were observed only in Maravilla del Mercado at six months of storage, with 50% less glucose than in cooked pulp (Figure 1). Starch and sugars are the main components of the dry matter (40–85%), and the source of glucose reported for both sweet potato and winter squash pulp [3,4,5,7,8,9]. Several authors have reported that in varieties of sweet potato and winter squash, losses of dry matter, total sugars, and glucose content for metabolism respiration and degradation during postharvest storage [6,7,9,18,19,20] were observed. Other authors have determined differences in starch granules as well as the amount and activity of enzyme α and β-amylases, which explain part of the different behaviors between varieties and the decreased rate of the total glucose content during storage time [6,9,11,18,21,22,23,24]. For winter squash, an increase in the total glucose content at the second month of storage was observed. This increase in glucose can be explained by the biosynthesis from other compounds and/or by a translocation from other parts of the fruit, as reported for watermelon [25] and melon [26].

### 3.2. In Vitro Bioaccessible Glucose Content in Cooked Pulp

The glucose release by in vitro digestion was higher in sweet potato (6.4 g) than winter squash cooked pulp (0.91 g). In sweet potato, the glucose released by digestion was between 8.4 and 5.1 g, without the effects of variety and storage time (Table 1).

According to these results, over time, storage caused less interference with other compounds of the pulp and probably provoked more access to enzymes in the site of action for the starch digestion. A change in the starch grains’ structure could be explained most easily by the digestion of the starch [8,9,18,19,20,21,22,23,27,28,29,30].

In winter squash cooked pulp, the glucose released by in vitro digestion depended on the interaction between variety and storage time (Table 1), with strong effects of variety. Atlas cooked pulp had 42–69% less glucose released by digestion than Maravilla del Mercado during every evaluated storage time. The highest content of glucose after digestion was obtained in the second month of storage in Maravilla del Mercado cooked pulp (1.4 g) and at the end of storage in Atlas (0.8 g) (Table 1). Similar to sweet potato, the type and properties of starch are different between species and varieties and can be modified with the storage time [4,6,11,20,28], but no major changes were detected in the total amount of glucose released by digestion.

**Figure 2:**
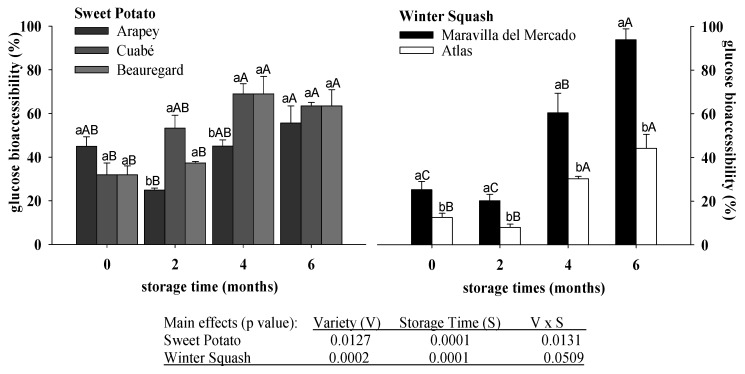
Percentage of in vitro bioaccessible glucose in cooked pulp from sweet potato and winter squash varieties stored for different times. Bars are means ± SEM (*n* = 6). For sweet potato or winter squash, lowercase letters on each column represent differences (Tukey *p* ≤ 0.05) between varieties at each storage time, and uppercase letters represent differences between storage times for each variety.

The percentage of glucose in vitro increased with the storage time in sweet potato (30–60%) and winter squash varieties (14–69%) (Figure 2). In sweet potato, the percentage of glucose bioaccessibility depended of the interaction of varieties and storage times. At the beginning and at the end of storage the percentage of bioaccessibility was similar between varieties, at an average of 37% at harvest and 59% at six months of storage. However, in the second month, Cuabé had twice the percentage of bioaccessible glucose than the others varieties (31%) (Figure 2). Despite the differences obtained in the total glucose content for sweet potato varieties and storage times, the cooking process was likely homogenized by the in vitro digestion. On the other hand, the winter squash pulp percentage of glucose bioaccessibility depended of the variety and storage time. It always showed a higher percentage in glucose bioaccessibility in Maravilla del Mercado than Atlas; at the same time, both varieties presented glucose bioaccessibility that rose two and three times after the second month of storage (Figure 2).

For all varieties of the sweet potato cooked pulp, the amount of released glucose by in vitro digestion in 100 g of cooked pulp (5.1 and 8.4 g) was greater than the total glucose content in the blood of healthy adults (70–99 mg dL^−1^, around 3.5–4.0 g of total glucose in blood) [31]. By contrast, in all cases for winter squash, cooked pulp was potentially only less than 25% of the total blood content.

## 4. Conclusions

The total glucose amount of sweet potato varieties was higher (14.5 g 100 g^−1^ fw) than winter squashes (4.0 100 g^−1^ fw). The Arapey sweet potato variety had the highest total content of glucose (21 g 100 g^−1^ fw) and winter squash varieties had the lowest (4 g 100 g^−1^ fw). These contents were affected by storage time, in which a prolonged storage of roots or fruits for more than two months caused a reduction in the level of total glucose. In sweet potato varieties, cooked pulp had lower glucose content than raw pulp after two months of storage time. However, in winter squash, cooking only affected the total glucose content in Maravilla del Mercado winter squash at the end of storage. The released glucose by in vitro digestion in sweet potato varieties studied here was similar and not affected by storage time, with amounts between 5.1 and 8.4 g for 100 g of cooked pulp. However, in winter squashes, Maravilla del Mercado had twice the amount of bioaccessible glucose than Atlas at every storage time (1.2 and 0.6 g 100 g^−1^ fw, respectively), and only the Maravilla del Mercado winter squash variety modified the released glucose by in vitro digestion with the storage time. Thus, the released glucose by in vitro digestion was low for both vegetables in relation to the carbohydrate daily requirement for adults (recommended dietary allowance 130 g day^−1^) [32]. Therefore, the sweet potato varieties studied here seem to be suitable for the recommended daily carbohydrate requirement for a healthy adult, and winter squash varieties could be recommended for people with a low tolerance of glucose in blood.

## Figures and Tables

**Table 1:** Total glucose (g) released by in vitro digestion in 100 g cooked pulp from sweet potato and winter squash varieties stored at different times.

Storage Time (Months)	Sweet Potato			Winter Squash	
	Arapey	Beauregard	Cuabé	Maravilla del Mercado	Atlas
0	8.4 ± 1.0	5.1 ± 0.8	5.2 ± 0.8	1.1 ± 0.03 ^a,B^	0.5 ± 0.07 ^b,A^
2	5.6 ± 0.3	5.3 ± 0.4	6.5 ± 0.6	1.4 ± 0.06 ^a,A^	0.4 ± 0.05 ^b,A^
4	6.5 ± 0.6	7.5 ± 1.4	7.3 ± 0.6	1.3 ± 0.01 ^a,A,B^	0.7 ± 0.07 ^b,A^
6	7.7 ± 0.6	6.0 ± 0.7	6.2 ± 0.2	1.1 ± 0.09 ^a,B^	0.8 ± 0.12 ^a,A^
Main Effects (*p* value): Variety (V) Storage Time (S) V × S					
Sweet Potato 0.1205 0.1958 0.0676					
Winter Squash 0.0001 0.0542 0.0032					

Means ± standard error (*n* = 6). For sweet potato or winter squash, lowercase letters on each row indicate differences (Tukey, *p* ≤ 0.05) between varieties in the same storage time, and uppercase letters indicate differences between storage times for each variety. No letters indicate a lack of a statistical difference.
